# An Information-Theoretic Characterization of the Optimal Gradient Sensing Response of Cells

**DOI:** 10.1371/journal.pcbi.0030153

**Published:** 2007-08-03

**Authors:** Burton W Andrews, Pablo A Iglesias

**Affiliations:** Department of Electrical and Computer Engineering, The Johns Hopkins University, Baltimore, Maryland, United States of America; California Institute of Technology, United States of America

## Abstract

Many cellular systems rely on the ability to interpret spatial heterogeneities in chemoattractant concentration to direct cell migration. The accuracy of this process is limited by stochastic fluctuations in the concentration of the external signal and in the internal signaling components. Here we use information theory to determine the optimal scheme to detect the location of an external chemoattractant source in the presence of noise. We compute the minimum amount of mutual information needed between the chemoattractant gradient and the internal signal to achieve a prespecified chemotactic accuracy. We show that more accurate chemotaxis requires greater mutual information. We also demonstrate that a priori information can improve chemotaxis efficiency. We compare the optimal signaling schemes with existing experimental measurements and models of eukaryotic gradient sensing. Remarkably, there is good quantitative agreement between the optimal response when no a priori assumption is made about the location of the existing source, and the observed experimental response of unpolarized Dictyostelium discoideum cells. In contrast, the measured response of polarized D. discoideum cells matches closely the optimal scheme, assuming prior knowledge of the external gradient—for example, through prolonged chemotaxis in a given direction. Our results demonstrate that different observed classes of responses in cells (polarized and unpolarized) are optimal under varying information assumptions.

## Author Summary

For many cell types, the direction of migration is determined in response to spatial differences in the concentration of chemoattractant, a process known as chemotaxis. Precise chemotaxis—that is, motility with low directional distortion—requires that cells make accurate decisions based on the stochastic fluctuations inherent in cell-surface receptor occupancy. Here, we use rate distortion theory, a branch of information theory, to determine chemotaxis strategies for cells based on this imperfect information about their environment. In engineering, rate distortion theory provides the information processing capabilities required to achieve a desired accuracy. We demonstrate that more accurate chemotaxis requires greater information. We also show that a priori information can improve chemotaxis efficiency. We compare the optimal signaling schemes to existing experimental measurements and models of eukaryotic gradient sensing and demonstrate that different observed types of cellular responses (polarized and unpolarized) are optimal under varying information assumptions. Our results also highlight the constraints that noise places on the performance of cellular systems.

## Introduction

Recently, there has been considerable research demonstrating the critical role played by random fluctuations in cellular signaling systems [1–3]. Stochastic variations are found in the external signaling molecules [4] as well as in the intracellular components [5]. They arise because of the small number of molecules involved in signaling and play significant roles in gene regulatory networks [6–10] as well as in prokaryotic [5,11] and eukaryotic signal transduction pathways [4,12,13].

The proper functioning of cellular signaling networks requires mechanisms that can tolerate the effects of noise [14]. However, questions remain as to how to evaluate the performance and efficiency of these cellular decision-making systems. How well does the signaling network of a cell make decisions based on the signaling cues available? Can improvements be made by altering the parameters or structure of the network? How efficiently are resources used? Here we argue that rate distortion theory [15], a branch of information theory, can be used to evaluate the effectiveness of such systems.

The application of information theory to the study of biology has been under way for some time [15–18] and has received considerable attention in the fields of neuroscience [19] and genetics [20–21]. However, the full breadth of this utility for biological signaling systems, in general, has not been realized, primarily because of the difficulty of defining “information” in general biological systems. Here we use rate distortion theory as a tool to study performance–cost tradeoffs in general spatial gradient sensing mechanisms, similar to those found in many eukaryotic cells, including neutrophils and amoebae. Rate distortion theory provides bounds on the rate at which information must be transmitted through a system to achieve a given performance criterion. Our results demonstrate that, depending on the prior knowledge that a cell has about its chemoattractant environment, different optimal chemotaxis strategies exist. Furthermore, we show that differences in the observed behaviors of unpolarized and polarized chemotactic cells correspond to these various optimally efficient decision-making processes.

## Results

To use rate distortion theory to determine optimal gradient sensing strategies, we develop a theoretical model of the cellular decision process. We define the system input Θ*_s_* to be a random variable that denotes the angle of the chemoattractant field (Figure 1A). In all notation that follows, random variables are denoted by capital letters, and lowercase denotes deterministic variables or realizations of random variables. Based on the perceived chemoattractant gradient, the cell responds by selecting an angle Θ*_r_*, which is a random variable that can represent either the location of intracellular markers of gradient sensing, such as phosphoinositide lipids [22] or the directional bias in which new pseudopods are generated [23,24]. The signal transduction network that generates *θ_r_* based on *θ_s_* incorporates binding and downstream signaling processes. It is modeled as the conditional probability distribution 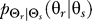 (Figure 1B).

**Figure 1:**
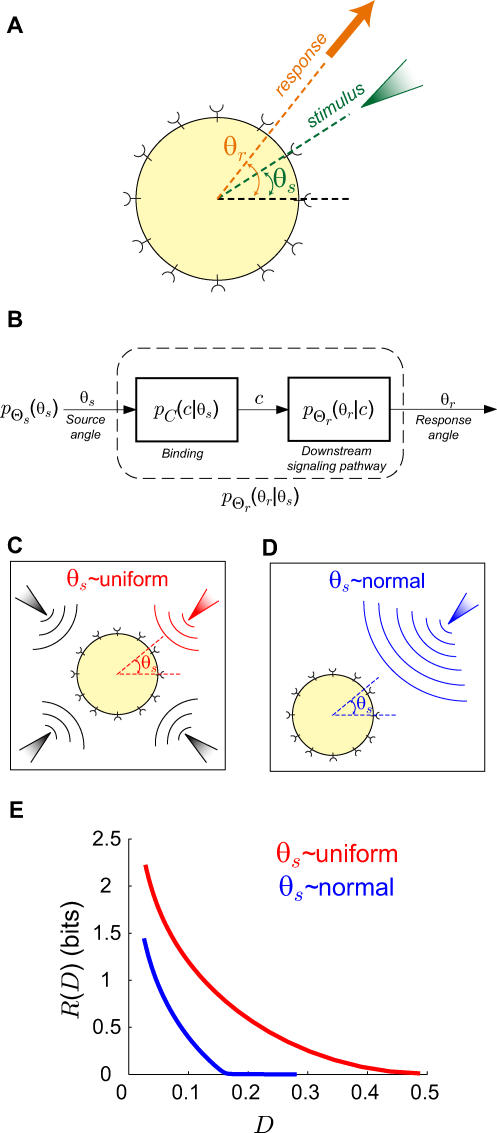
Gradient Sensing Model and Chemoattractant Gradient Distributions (A) We assume that cells are exposed to a linear gradient coming from a randomly chosen angle Θ*_s_* and respond by localizing intracellular markers or extending pseudopods at an angle Θ*_r_*. (B–D) The actual response angle Θ*_r_* = *θ_r_* is determined by the conditional probability distribution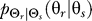 (stimulus–response map). We later assume this map to consist of a ligand-receptor binding component that yields ligand-bound receptor complexes *C* and a downstream signaling component (Materials and Methods). Two classes of gradient angle distributions are assumed: uniform (C) and normal (D). The former is appropriate to describe cells that have been newly introduced to a gradient or cells that are experiencing rapid changes in the location of the chemoattractant source. A normal distribution is suitable for cells that have experienced stimulation by and chemotaxis toward a source of attractant—such as seven-hour D. discoideum cells that are in a stream [22]. (E) Shown are the rate distortion functions for the uniform (red) and normal (blue) source distributions with a mean and variance for the normal distribution of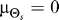 and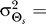 45°, respectively. The rate distortion curve (*R*(*D*)) represents the minimum amount of information (units of bits) required to meet a given distortion requirement (*E*[*d*] ≤ *D*). For a given *D*, the mutual information curve between signal and response for any practical scheme must lie above this limit.

We assume that Θ*_s_* arises from one of two classes of source distributions (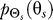 ). The first class assumes that Θ*_s_* is uniformly distributed and is representative of a cell with no a priori directional bias. This distribution would be expected in “naïve” cells that have no previous exposure to chemoattractant gradients (Figure 1C). The second, a normal distribution with mean 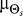 and variance 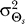 , represents biased cells that expect gradients to come predominantly from a predetermined direction (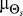 ) (Figure 1D). This bias may correspond to that of cells that have experienced prolonged exposure to a chemoattractant source in a fixed direction.

Because the goal of a chemotaxing cell is to navigate (Θ*_r_*) in the direction of greatest ligand concentration (Θ*_s_*), we characterize the quality of this decision with a distortion function that is related to the chemotactic index:

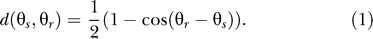



The distortion function is zero—that is, there is no distortion—when the directional response of the cell is aligned with the gradient, and one when the two differ by 180°.

Using this model, we computed the rate distortion function (*R*(*D*)) (Figure 1E, Materials and Methods). This function describes the minimum amount of mutual information (units of bits) between stimulus and response required for the cell to achieve a given expected distortion (*E*[*d*] ≤ *D*) when making a gradient sensing decision. For both classes of distributions, the rate distortion function is a decreasing function of required fidelity. Moreover, the rate distortion curve for a normal distribution lies below that for the uniform source distribution for all values of *D*. Thus, more information is needed to achieve a required level of fidelity when the cell has no a priori knowledge of the chemoattractant direction.

To investigate further the differences in cellular response to the two classes of a priori knowledge, we computed the map 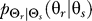 that yields a given point on the rate distortion curve. These maps represent the optimal signaling scheme for any required fidelity (Figure 2). For naïve cells, the optimal 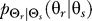 is characterized by a distribution whose peak is at the angle of the external gradient and whose spread is determined by the required fidelity. More precise chemotaxis (smaller *D*) requires sharper distributions (Figure 2A–2D).

**Figure 2:**
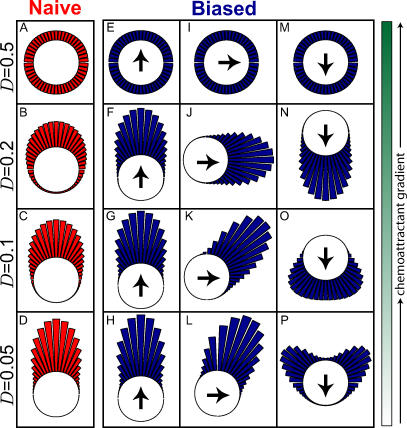
Optimal Gradient Sensing Performance Circular histogram describing the stimulus–response map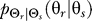 that achieves points *R*(*D*) on the rate distortion curve for various required levels of distortion (*D* = 0.5, 0.2, 0.1, and 0.05). The length of each line is proportional to the probability that the cell will respond in the direction shown. All panels assume that the chemoattractant gradient is toward the top of the figure. (A–D) Describe the response of cells that are optimized for a uniform source distribution. (E–P) Depict the response of cells optimized for a normal source distribution where the mean is indicated by the direction of the black arrow inside the circle and the variance is 45°. In (E–H) the internal cue is co-aligned with the gradient; in (I–L) they are 90° apart; in (M–P) they are 180° apart.

For cells that have an a priori bias, the optimal 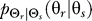 depends on the allowable distortion and the degree to which the gradient is co-aligned with the bias direction (Figure 2E–2P). For large allowable distortions (*D* = 0.2), essentially no information about the stimulus is required, and a distribution 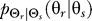 centered at the direction of bias and independent of the chemoattractant gradient is sufficient (Figure 2F, 2J, and 2N). As with naïve cells, the optimal response becomes uniform as the allowable distortion increases (Figure 2E, 2I, and 2M). However, for more stringent requirements (smaller *D*), the optimal decision is more narrowly distributed—particularly when the gradient and bias angle are co-aligned. When these two angles are not aligned, the optimal decision process follows a complex function of the gradient and internal bias angles. For large allowable distortion, the decision tends to point toward the angle of the preexisting bias (Figure 2J, 2N, and 2O). However, as the required fidelity becomes more stringent, the optimal decision increasingly reflects the direction of the chemoattractant gradient (Figure 2K, 2L, and 2P). In particular, when the bias and gradient angle are misaligned by 180°, the optimal response for stringent distortions is almost opposite the bias (Figure 2P). As *D* decreases even further, the optimal response approaches complete alignment with the gradient (unpublished data).

We next determined the effect of the optimal stimulus–response maps on chemotaxing cells through simulation (Figure 3, Materials and Methods). Cells in a 2-D environment were exposed to a ligand source at a fixed location, and their final position after chemotaxing for 400 s was observed (Figure 3A). Both naïve and biased cells chemotax more efficiently (*p* < 10^−9^ using Student's *t*-test) if their decision process is optimized for more stringent chemotactic index requirements (smaller distortion). Moreover, cells that employ the strategy optimized under a preexisting bias chemotax better (*p* < 10^−9^) than those whose decision strategy is optimal for a uniform source distribution.

**Figure 3:**
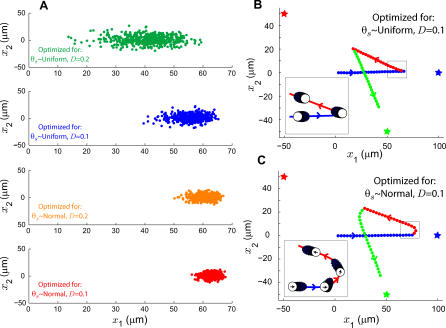
Chemotaxis Simulation for Cells Optimized under Various Gradient Assumptions (A) For each simulation, cells were initially at the origin (0,0) of a 2-D environment and exposed to a point source of attractant 100 μm to the right. Chemotaxis was simulated for 400 s and the cells' final position noted. Shown is the scatter plot of the final position of 500 cells employing the optimal stimulus–response map for a uniform source distribution with average distortion *D* = 0.2 (green), uniform source with *D* = 0.1 (blue), normal source with *D* = 0.2 (orange), and normal source with *D* = 0.1 (red). Final distance from the source for naïve cells: 60 ± 8 μm for *D* = 0.2, 45 ± 4 for *D* = 0.1; and 40 ± 3 for *D* = 0.2 versus 37 ± 2 for *D* = 0.1, for biased cells. (B,C) Mean trajectory of simulated cells employing the optimal stimulus–response map for uniform (B) and normal (C) source distributions with average distortion *D* = 0.1. Cells were exposed to an attractant source at location (100,0) at time 0 s (blue star) followed by a source at location (−50,50) at 500 s (red star) and (50, −50) at 900 s (green star). The dots indicate the average of 500 cell trajectories. The insets show the stimulus–response maps, as in Figure 2, at several points before and after a change in gradient source. For all simulations, position units are μm, cell velocity is 10 μm/min, and biased cells are optimized for a variance of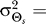 45°.

The simulations above demonstrate the advantage of having a biased distribution when this is co-aligned with the gradient. To explore the effect of an inaccurately biased assumption, we computed the average trajectory of cells in the presence of a source that changes location with time. Cells optimized for uniform source distributions change direction immediately in response to changes in attractant location (Figure 3B). However, cells that are optimal for a preexisting bias respond initially by continuing to move in the direction of the previous gradient and change direction in a more gradual manner (Figure 3C).

The distributions of Figure 2 provide the optimal strategies given a required minimal chemotactic efficiency. To compare these theoretically optimal performances with the signaling pathway of chemotaxing cells, we used a local-excitation, global-inhibition (LEGI) model previously proposed to explain the gradient sensing pathway of D. discoideum [25–27]. The model differs from previous versions in that the response subtracts the minimum receptor occupancy (Materials and Methods). This model is also parameterized by a Hill coefficient that describes the degree of amplification, relative to the external gradient, observed in the cell's response [28]. Experimentally, Hill coefficients between 3 and 7 have been observed in the chemoattractant-mediated asymmetric localization of phosphoinositides in D. discoideum and neutrophils [27–29].

We computed the expected distortion and mutual information for the stimulus–response map characterized by the LEGI model for a range of Hill coefficients under both uniform and normal source distributions (Figure 4A and 4B). For comparison with the optimal lower bound provided by the rate distortion curve, these were plotted as points on the same distortion–information plane as *R*(*D*) (Figure 4C and 4D). For both types of distributions, increasing the Hill coefficient decreases expected distortion. Remarkably, for the uniform case, all points lie on the rate distortion curve (Figure 4C), suggesting that cells employing a LEGI mechanism to guide directional decisions are optimized to minimize the expected distortion for a given information constraint under uniform source distributions. For normally distributed gradients, the points lie above the rate distortion curve (Figure 4D) implying less than optimal information processing. However, the degree of non-optimality decreases with increasing Hill coefficient.

**Figure 4:**
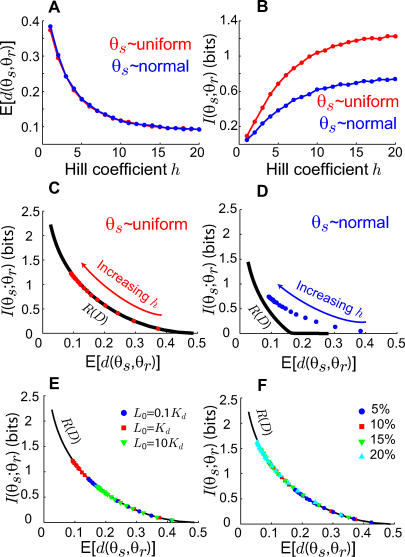
Achievable Performance of the LEGI Model of Gradient Sensing (A) Expected distortion of the LEGI model for various degrees of amplification (characterized by an effective Hill coefficient *h*) when the probability distribution of the direction of the attractant gradient is uniform (red) and normal (blue). (B) Mutual information between stimulus and response for a uniform (red) and normal (blue) source distribution for varying Hill coefficients. (C–D) Superimposed plots of the best achievable rate (black line) as well as the LEGI model (red and blue circles) for varying Hill coefficients (*h*) assuming either uniform (C) or normal (D) source distributions. (E–F) Expected distortion versus mutual information for various Hill coefficients (*h*) under different chemoattractant gradients and a uniformly distributed gradient angle. In (E) the gradient is 10% and the midpoint concentration is 0.1*K_d_* (blue circles), *K_d_* (red squares), and 10 *K_d_* (green triangles). In (F) the midpoint concentration is *K_d_* and the gradient is ±5% (blue circles), ±10% (red squares), ±15% (green triangles), and ±20% (light blue triangles). Parameters used in (A–D) are *N* = 50, *a* = 220, *b* = 20, *K_d_* = 200,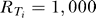 for all *i*,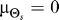 , and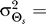 45°.

To investigate the effect of the ligand profile on decision-making performance, we repeated the above computations for different mean levels of chemoattractant and gradient. We first fixed the gradient (±10% across the radius of the cell) and varied the mean level of chemoattractant (Figure 4E). The least amount of distortion and highest mutual information was found for ligand concentrations around *K_d_*. At lower chemoattractant concentrations, the binding noise dominates; at higher concentrations, receptor saturation reduces the cell's ability to sense the gradient. As expected, when the mean level of chemoattractant was fixed (at *K_d_*), the expected distortion decreased and the mutual information increased for higher gradients (Figure 4F). Interestingly, in all cases, the performance of the LEGI model lies along a single curve in the distortion–information plane—the effects of noise and variations in other system parameters simply slides the performance point along this curve (Figure 4E–4F).

The above results demonstrate that the output distributions of the LEGI mechanism match those optimal for uniformly distributed gradient angles. To examine how this signaling mechanism may be modified to account for biased input–output maps (i.e., those optimal for normally distributed gradient angles), we combined gradient amplification from the LEGI model with a previously proposed model of directional sensing that incorporates an internal bias [13] (Materials and Methods). In this model, the cell responds to a signal that combines the external gradient and an internal cue that has a spatial predisposition toward an angle *θ_b_*. The degree to which *θ_b_* contributes is quantified by a parameter *ɛ*. The value of *ɛ* represents the extent to which the cell is biased toward a given location. As *ɛ* increases, the contribution of the internal bias increases and the most probable response angle moves closer to *θ_b_*.

We computed the response of this modified LEGI model to a chemoattractant source under both unbiased (*ɛ* = 0) and biased (*ɛ* ≠ 0) assumptions for a variety of *ɛ* and *h* (unpublished data) and compared the responses with the optimal signaling maps. The optimal response for uniformly distributed source angles was captured well by the model with no bias (*ɛ* = 0). This optimality of the unbiased LEGI mechanism was seen previously (Figure 4C), and the expected distortion for which the model is optimal is dependent upon *h*. For example, *ɛ* = 0, and a Hill coefficient of *h* = 4.8 yields a model response that closely matches the optimal response of an unbiased cell with an allowable distortion of *D* = 0.1 (Figure 5A).

**Figure 5:**
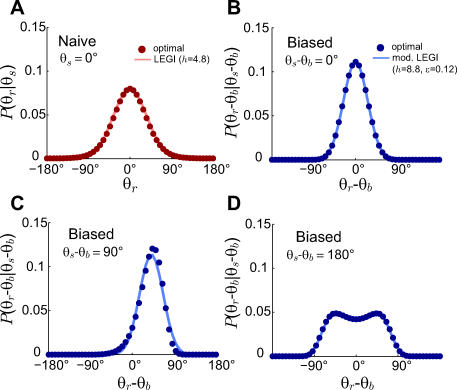
Comparison of Modified LEGI Model to the Optimal Response Distribution (A) Probability distribution of the response to a gradient angle of *θ_s_* = 0 that optimally achieves the rate distortion curve with *D* = 0.1 for Θ*_s_* uniformly distributed (dark red circles). Also shown is the response to *θ_s_* = 0 of the LEGI model with *h* = 4.8 (light red line). (B–D) Probability distribution of the optimal response for *D* = 0.1 to a normally distributed input with variance 45° and an observed gradient angle that differs from the mean (bias) by 0°, 90°, and 180° (dark blue circles in (B), (C), and (D), respectively). Also shown is the response of the modified LEGI model with *ɛ* = 0.12 and *h* = 8.8 (light blue lines). In all panels (A–D), binding is excluded from the computations of the model response (i.e., *Y_i_ = L*(*θ_i_*)) so as to more accurately compare Hill coefficients to experimental observations. Inclusion of binding also yields good fits to the optimal response, only with slightly larger Hill coefficients.

The optimal response to normally distributed source angles was captured well by the modified LEGI model with nonzero bias (*ɛ* ≠ 0). The angle variance 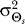 and allowable distortion *D* for which the model was optimal is dependent on *h* and *ɛ*. The optimal response for *D* = 0.1 and a normally distributed source angle with 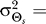 45° is matched well by the model when *ɛ* = 0.12 and *h* = 8.8 (Figure 5B–5D). Note that when the gradient is aligned with the bias and *D* is fixed, the apparent amplification for biased cells is larger than that for naïve cells (Figure 5A–5B). This is consistent with experimental data which shows that polarized cells have higher amplification than unpolarized cells [28].

## Discussion

A common challenge among cellular signaling systems is making decisions based on noisy chemical cues that offer incomplete information about the environment. This is particularly true for chemotaxing cells such as D. discoideum and neutrophils that must determine the direction of a chemical gradient. Accurate gradient sensing is hindered by diffusive fluctuations in the concentration of chemoattractant as well as random variations inherent in the dynamics of the signaling process [30,31]. Here we use rate distortion theory as a quantitative framework within which to study the effectiveness of gradient sensing strategies with respect to underlying costs and performance objectives. Rate distortion theory gives the strategy that minimizes the information that must be acquired about the gradient direction to satisfy given performance constraints. Because the input (gradient angle) is noisy, zero distortion is not possible. We use this optimal strategy as a benchmark with which to compare responses observed in chemotaxing cells.

Our results help explain differences in observed behavioral responses of polarized and unpolarized D. discoideum cells [22]. D. discoideum cells acquire the ability to chemotax to external cAMP gradients approximately four hours after starvation. Initially, cells show uniform sensitivity to chemoattractant signals around their perimeter. Moreover, when exposed to changes in the gradient, these unpolarized cells respond by redistributing intracellular markers rapidly. The unpolarized behavior can be induced through the addition of chemicals, such as Latrunculin A, that inhibit actin polymerization but leave intact the ability to detect spatial gradients and respond by redistributing several internal markers [27,28].

Using rate distortion theory, we showed that many of these characteristics can be obtained as optimal strategies for cells that make no a priori assumption on the direction of the external gradient. In particular, cells that assume uniformly distributed gradients show uniform sensitivity (Figure 2A–2D) and change direction rapidly when the chemoattractant field changes (Figure 3B). Previously, it has been shown that the cAMP response of unpolarized cells is closely captured by the LEGI model. Our results show that, when the minimum receptor signal is subtracted everywhere (Materials and Methods), the LEGI mechanism is optimal in terms of the rate distortion function for cells that have no a priori knowledge of their environment (Figures 4 and 5A). Without the subtraction, the matches between model and optimal response are not as good and require unreasonably high Hill coefficients. The subtraction can represent an internally derived threshold such as has been observed experimentally [28] and previously modeled [32].

After a prolonged period of directed motion to an existing gradient, D. discoideum cells become increasingly polarized, acquiring distinctive leading and trailing edges. The anterior portion of these cells is more sensitive, so that cells that experience a change in the chemoattractant environment will turn toward the new direction of gradient. This polarity can be changed, however, by steep gradients [22]. We are able to capture these observed behaviors using rate distortion theory by assuming that the cell has a priori knowledge of the direction of the chemoattractant gradient. This assumption would be reasonable for cells, such as D. discoideum cells starved for seven hours, that have been chemotaxing in a given direction for several hours (so-called streaming cells). Cells whose signaling scheme was optimal with respect to normally distributed gradients respond in directions that take this prior knowledge into account (Figure 2E–2P) and are more efficient than cells that make no such a priori assumption (Figure 3A). When the angles of the preexisting bias and chemoattractant field agree, the optimal signaling schemes show greater amplification of the external gradients than those where no a priori assumption is made (Figure 2B–2D versus Figure 2F–2H), as has been observed experimentally in cellular responses [13,28]. This suggests that, to tolerate a given level of distortion, the presence of prior (correct) knowledge allows the cell to follow the gradient more aggressively. These biased signaling schemes also display turning behavior to changes in gradient location (Figure 3C). Moreover, the degree to which the preexisting bias dominates the response can be overcome by either more stringent distortion requirements (Figure 2O versus Figure 2P), or by stronger external gradients (unpublished data). The optimal signaling response of these systems is matched accurately by a modified LEGI mechanism [13] that allows for an internal cue (Figure 5B–5D). In *D. discoideum,* this internal directional cue may be effected by asymmetric distributions of G-proteins [33]. Other cells, such as S. cerevisiae may rely on receptor redistribution [34].

Our results demonstrate that demanding higher fidelity (lower distortion values) leads to improved chemotaxis (Figure 3). Lower values of *D* require greater amplification of the external chemoattractant gradient in the optimal signaling scheme (Figure 2) and are recovered by the LEGI model with higher Hill coefficients (Figure 4). For cells with no a priori bias, *D* = 0.1 and 0.2 require Hill coefficients of 4.8 (Figure 5A) and 2.0 (unpublished data), respectively. Experimental quantification of this amplification in both unpolarized D. discoideum cells (3.1 ± 0.89) and neutrophils (3.25 ± 2.0) suggests that the signaling mechanism has been optimized for a value of *D* ≈ 0.15 (Figure 6A [28,29]). For biased cells, the optimal response depends not only on the allowable distortion *D*, but also on the variance 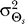 , which is a reflection of how certain the cell is of the gradient angle. For optimal gradient sensing, a higher degree of certainty (smaller 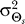 ) must be matched by a larger emphasis on the internal bias signal in the model (larger *ɛ*) (Figure 6B). Furthermore, less stringent allowable distortions require less information about the gradient angle and thus are achieved with a larger emphasis on the bias (larger *ɛ*).

**Figure 6:**
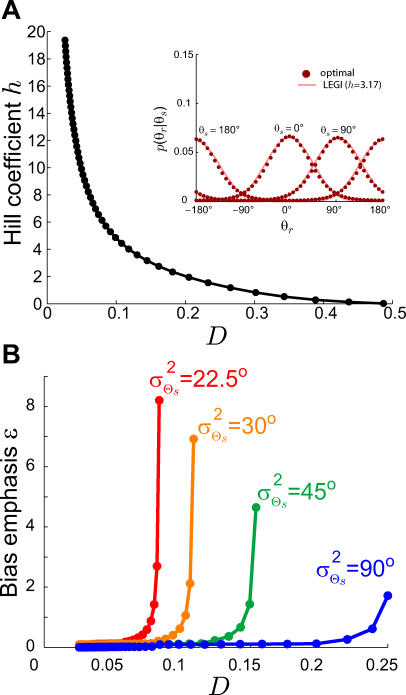
Optimal Model Parameters (A) Plotted is the Hill coefficient *h* that best fits the LEGI model to the optimal response function that achieves *R*(*D*) for Θ*_s_* distributed uniformly and varying *D*. The optimal response for a distortion of *D* ≈ 0.15 is best fit by the LEGI model with a Hill coefficient of *h* ≈ 3.17 (inset), which is close to that observed in unpolarized D. discoideum cells (3.1 ± 0.89) and neutrophils (3.25 ± 2.0) [28,29]. (B) The value of *ɛ* for which the response of the modified LEGI model matches the optimal response depends on the allowable distortion *D* and the gradient angle variance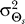 *.* Larger *ɛ* (larger contribution of the internal bias signal *b* to the overall response) is required for smaller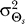 and larger *D*. As in Figure 5, binding is excluded from the model response.

As lower values of *D* would lead to more efficient chemotaxis (Figure 3), it is worth asking why smaller values are not seen experimentally. Our results do not consider the cost associated with demanding more accurate fidelity. To achieve lower distortion values requires that the cell have increasingly greater information about the chemoattractant environment (Figure 4B). However, obtaining this information is costly. In engineering systems, this information cost is quantified by the number of bits of information that must be processed. How to quantify information in a biological system is less clear, though the likely measure is based on energetic costs. Energy has been previously suggested as a means of measuring the cost of information [16,17] and some progress has been made in this area in neurobiology [35,36]; however, the energetic costs associated with more general cellular signaling mechanisms are less certain. Recent analysis reveals that increasing amplification, either through allosteric cooperativity or covalent modification, requires larger free energy [37]. Coupled with our findings that lower distortion requires greater amplification (higher Hill coefficient) to achieve greater mutual information between stimulus and response, these results highlight that the improving chemotactic performance comes with increasing costs.

The feasibility of using the rate distortion framework to evaluate the decision-making performance of a particular biological signaling network depends primarily on the ability to characterize the signaling source distribution and the distortion function. Specifying a distortion function requires a quantitative description of a specific goal for the cell, which is not always possible. However, performance analysis of various distortion functions may reveal which objectives the cell has evolved to best address. Also, it is often reasonable to assume that the signaling mechanisms of at least simple cellular systems balance performance–cost tradeoffs nearly optimally [14,38]. In light of this, rate distortion theory is a useful tool in providing insight into the types of decision-making processes that cellular systems are likely to utilize.

## Materials and Methods

### Model.

The gradient sensing system detects an input gradient signal *θ_s_* that arises from a probability distribution 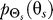 . The cellular signaling network, described by the conditional distribution 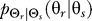 , yields an output *θ_r_* that indicates the direction of cell movement in response to the gradient. The distortion function 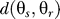 is a measure of how well the response is aligned with the gradient. Smaller distortions represent better alignment.

### Rate distortion.

The entropy, 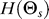 , of the random variable Θ*_s_* characterizes the amount of uncertainty in the gradient direction: 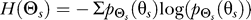 . The mutual information *I*(Θ*_s_*; Θ*_r_*) between Θ*_s_* and Θ*_r_* is the reduction in uncertainty of the gradient direction given the response direction. Mathematically,

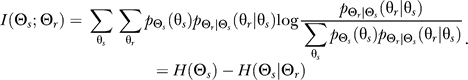



The rate distortion function gives the minimum rate *R*(*D*) (with units of bits) at which data must be transmitted through the system so that the expected distortion is no greater than some preset level *D*; i.e., *E*[*d*(Θ*_s_*,Θ*_r_*)] ≤ *D* for a given *D* and a given input distribution 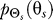 . It is known that [39]

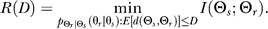



While the term “rate” here is not clearly defined in the context of the biological system, we can view rate distortion as providing a lower bound on the information processing of downstream cellular networks required to achieve certain performance criteria.

### Computation of *R*(*D*).

In general, the rate distortion function is given by

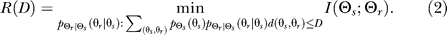
and can be solved using the method of Lagrange multipliers [39]. Theoretical solutions are well known for common source distributions and distortion functions such as Gaussian and squared error, respectively. However, in our system an analytical solution is intractable, so we solve the problem computationally using the Blahut-Arimoto algorithm described in Chapter 13 of [39]. The basic steps are as follows: we assume a particular marginal output distribution 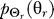 . In our computations, we initialize 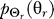 as a uniform distribution. We then compute the conditional distribution 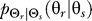 that minimizes the mutual information subject to the distortion constraint. Next, given 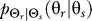 , we compute the marginal distribution 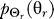 that minimizes the mutual information. We then repeat these last two steps until convergence. The limiting mutual information as these steps are carried out has been shown to be *R*(*D*) where *D* is determined by the Lagrange multiplier chosen in the minimization.

### Computation of I(Θ_s_; Θ_r_).

In Figure 4, estimation of the mutual information between the gradient angle Θ*_s_* and the cell's directional response Θ*_r_* is computed as follows. First, 100,000 independent instances of the input are generated in Matlab version 7.3 (Mathworks, http://mathworks.com) according to the source distribution. Then, after computing the corresponding bound-receptor concentration (*c*) for each input, a random output is generated for each input via the distribution 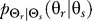 . Given each input–output pair, the mutual information is estimated using the Matlab command information.m, developed by R. Moddemeijer [40].

### Chemotaxis simulation.

We simulated a cell chemotaxing toward a point source of attractant in a 2-D environment in Matlab. Define the position of the cell and source at time step *k* as (*x*_1_(*k*),*x*_2_(*k*)) and (*s*_1_(*k*),*s*_2_(*k*)), respectively. At each simulation time step, the cell measures *θ_s_*(*k*), the source angle, as tan^−1^[(*s*_2_(*k*) – *x*_2_(*k*))/(*s*_1_(*k*) – *x*_1_(*k*))]. The direction of cellular movement is then randomly generated based on the input–output map 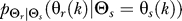 , and the cell's position at time step *k* + 1 is computed as

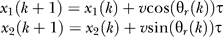
where *v* is the velocity of the cell and τ is the size of the simulation time step in seconds.

The input–output map 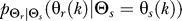 is computed as the optimal conditional distribution that achieves the point (*D*,*R*(*D*)) on the rate distortion curve with *D* = 0.1 for both uniform and normal source distributions 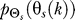 (Figure 2). For the case of a normal source distribution, the optimal 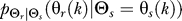 at time *k* is computed assuming Θ*_s_* has mean 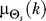 (the bias) and variance 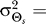 45°, where 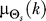 is the average of the six previous input values (two minutes of data). To avoid periodicity complications, we compute 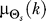 as

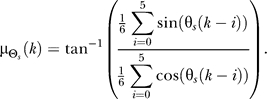



This adjustment of 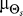 over time simulates a realignment of the cell's a priori presumption of the source location as time progresses. With no adjustment, a cell would continue to bias its directional decision toward a given source location, even after that source has moved, and successful chemotaxis would not occur. Parameters used are *τ* = 20 s and *ν* = 10 μm/min.

### Gradient sensing model of D. discoideum.

Our model is based on previously published models [13,26,27]. In these cells, ligand concentration is sensed by receptors that are found uniformly distributed along the membrane of the cell. We divide the cell into *N* sectors, *θ_i_*, *i* ∈ (1, … , *N*}, and assume that receptors in each sector interact independently with ligand molecules in the vicinity of the sector. The discrete random variable Θ*_s_* denotes the angle of greatest ligand concentration (the gradient or source angle) and takes values on the set {*θ*_1_, … , *θ*_N_}. Assuming a linear gradient of ligand in the environment and a particular gradient angle Θ*_s_* = *θ_s_*, the ligand concentration in the vicinity of sector *θ_i_* is *L*(*θ_i_*) = *a* − *b*(1 − cos (*θ_i_* − *θ_s_*)), where *a* and *b* are the maximum ligand concentration experienced by the cell and the size of the gradient, respectively.

Binding is assumed at steady state so that, given the gradient direction Θ*_s_* = *θ_s_*, the probability distribution (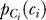 ) of bound receptor complexes (*C_i_*) in sector *i* is normal with mean μ*_C_i__* = 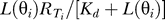 and variance 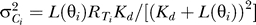 , where 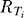 is the total receptor concentration in sector *i* and *K_d_* is the ligand-receptor binding dissociation constant, assumed to be the same for all sectors [41]. Unless otherwise specified, we use *N* = 50, *K_d_* = *L*_0_, where *L*_0_ is the ligand concentration at the center of the cell and 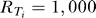 for all *i* [31,42].

Following the work of Samadani et al. [13], which is based on a phenomenological model, we assume the response of the cell in sector *i* is characterized by an effective signal *Y_i_*, which combines the effect of the external gradient and an internal bias: *Y_i_* = *C_i_b_i_*. The internal bias is given by:

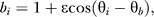
where the angle *θ_b_* represents the direction of the internal bias, and the variable *ɛ* is the magnitude of the internal bias contribution. When *ɛ* = 0, the cell is unbiased.

To relate the signal *Y_i_* to the probability of moving in a particular direction, we employ a LEGI mechanism [25–27]. In this mechanism, the response signal in a given direction is determined by the ratio of the local signal (*Y_i_*) to a global signal that is representative of the mean level of local signal (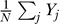 ). We model the effect of amplification phenomenologically through a Hill coefficient, so that the response at a location *θ_i_* along the membrane equals

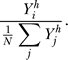



Fitting this model to match the optimally derived responses leads to extremely large Hill coefficients (*h* > 20). Better matches that also require smaller Hill coefficients are found when we subtract the minimum before amplification, so that the response at angle *θ_i_* is:

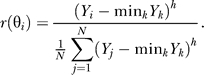
This minimum subtraction mimics observed responses of cells that suggest that there is no response at the rear of the cell [28].

Finally, normalization leads to the conditional probability distribution between bound-receptor concentration and the response angle given by:

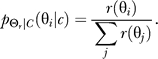



## Abbreviations

LEGI: local-excitation, global-inhibition
